# A novel bifunctional aldehyde/alcohol dehydrogenase catalyzing reduction of acetyl-CoA to ethanol at temperatures up to 95 °C

**DOI:** 10.1038/s41598-020-80159-7

**Published:** 2021-01-13

**Authors:** Qiang Wang, Chong Sha, Hongcheng Wang, Kesen Ma, Juergen Wiegle, Abd El-Fatah Abomohra, Weilan Shao

**Affiliations:** 1 School of the Environment and Safety Engineering, Biofuels Institute, Jiangsu University, Zhenjiang, 212013 Jiangsu China; 2 Department of Biology, University of Waterloo, Waterloo, ON N2L 3G1 Canada; 3 Department of Microbiology, University of Georgia, Athens, GA 30602 USA; 4 Department of Environmental Engineering, School of Architecture and Civil Engineering, Chengdu University, Chengdu, 610106 China; 5 Botany Department, Faculty of Science, Tanta University, Tanta, 31527 Egypt

**Keywords:** Biochemistry, Biotechnology, Microbiology

## Abstract

Hyperthermophilic *Thermotoga* spp. are excellent candidates for the biosynthesis of cellulosic ethanol producing strains because they can grow optimally at 80 °C with ability to degrade and utilize cellulosic biomass. In *T. neapolitana* (*Tne*), a putative iron-containing alcohol dehydrogenase was, for the first time, revealed to be a bifunctional aldehyde/alcohol dehydrogenase (Fe-AAdh) that catalyzed both reactions from acetyl-coenzyme A (ac-CoA) to acetaldehyde (ac-ald), and from ac-ald to ethanol, while the putative aldehyde dehydrogenase (Aldh) exhibited only CoA-independent activity that oxidizes ac-ald to acetic acid. The biochemical properties of Fe-AAdh were characterized, and bioinformatics were analyzed. Fe-AAdh exhibited the highest activities for the reductions of ac-CoA and acetaldehyde at 80–85 °C, pH 7.54, and had a 1-h half-life at about 92 °C. The Fe-AAdh gene is highly conserved in *Thermotoga* spp., *Pyrococcus furiosus* and *Thermococcus kodakarensis*, indicating the existence of a fermentation pathway from ac-CoA to ethanol via acetaldehyde as the intermediate in hyperthermophiles.

## Introduction

As a leading candidate among alternatives to petroleum-derived transportation fuels, the production of fuel ethanol from cellulosic biomass has received considerable attention and effort over the last two decades^1,2^. However, the high costs of converting biomass to sugars and distilling ethanol at low concentrations from fermentation products are the primary factors that impede the establishment of a cellulosic biofuels industry^1,3^. Thermophiles can produce cellulosic ethanol at a high temperature, where ethanol is directly distillated from fermentation, and biodegradation of lignocellulose can be simultaneously achieved when these thermophiles carry and express cellulase and hemi-cellulase genes^3,4^.

Thermophiles can be categorized into three groups based on their optimal growth temperatures: moderate thermophiles grow optimally between 50 and 64 °C; extreme thermophiles and hyperthermophiles can grow optimally between 65 and 79 °C and above 80 °C, respectively^5,6^. *Thermotoga* spp. are cellulolytic and hemicellulolytic hyperthermophiles growing at temperatures up to 90 °C. They are excellent candidates for the construction of a cellulosic ethanol producer. At present, research has been focused on the identification of hyperthermophilic enzymes for an effective ethanol fermentation pathway in hyperthermophiles.

Generally, the final steps of ethanol fermentation in anaerobes are two reversible redox reactions, from ac-CoA to acetaldehyde (ac-ald) catalyzed by the CoA-dependent aldehyde dehydrogenase (Aldh) (enzyme classification number, EC 1.2.1.10), and from ac-ald to ethanol catalyzed by alcohol dehydrogenase (Adh, enzyme classification number, EC 1.1.1.2). Generally, Aldhs could be divided into two types based on their dependence on CoA; the CoA-independent Aldhs (EC 1.2.1.3) and the CoA-dependent Aldhs (EC 1.2.1.10). The CoA-independent Aldhs catalyze the irreversible oxidation of aldehydes to their corresponding acids^7^. However, CoA-dependent Aldhs catalyze the reversible conversion of acyl-CoA to the corresponding aldehydes^8^. Among the extremely thermophilic microbes, *Thermoanaerobacter ethanolicus* (*Tet*) grows optimally at 69 °C, and produces ethanol as a main fermentation product. The ethanol fermentation pathway of *Tet* is established by AdhA, AdhB, and AdhE, where AdhB and AdhE are bifunctional aldehyde/alcohol dehydrogenases (AAdh)^9,10^. However, to our knowledge, CoA-dependent Aldh activity has not yet been reported in any hyperthermophiles such as *Thermotoga* that grows at temperature up to 90 °C. In *Thermotoga maritima* (*Tma*) and *Thermotoga hypogea* (*Thy*), the pyruvate ferredoxin oxidoreductase (POR or PFOR) has CoA-dependent pyruvate decarboxylase (PDC) activity^11,12^. However, the bifunctional POR/PDC exhibited a very low specific activity of PDC, which was less than 2% of that of POR activity^11,12^. These results revealed that more than 98% of pyruvate would be converted to ac-CoA by POR, and ac-CoA would be further converted to acetic acid in the absence of an enzyme to catalyze the reduction of ac-CoA to produce ac-ald.

In our previous work, it has been revealed that the CoA-dependent Aldh activity can be performed by an enzyme that is annotated as alcohol dehydrogenases^8,10,13^. We think it necessary to characterize all the enzymes encoded by the alcohol dehydrogenase genes in a hyperthermophilic strain to search a CoA-dependent Aldh activity that is possibly occurring in hyperthermophilic species of *Thermotoga*. In this paper, we report gene cloning and expression, enzyme purification and characterization of a novel bifunctional iron-containing aldehyde/alcohol dehydrogenase (Fe-AAdh) from *Thermotoga neapolitana* (*Tne*). Furthermore, the similarity and distribution of Fe-AAdh in other microbes are also discussed basing on the analysis of bioinformatics. Knowledge about Fe-AAdh of *Tne* will be beneficial for further understanding an ethanol fermentation pathway in hyperthermophiles.

## Materials and methods

### Bacterial strains and plasmids

*Tne* strain NS-E (DSM 4359) was purchased from the German Collection of Microorganisms and Cell Cultures (DSMZ). The organism was grown anaerobically in *Tma* basal medium (TBM) with 0.5% glucose at 80 °C^14^.

*Escherichia coli* JM109 (Promega) was used as host for cloning and expression of genes in a pHsh vector (GenBank accession number: FJ571619.1), constructed by Shine E Biotech, Nanjing, China. *E. coli* cells were routinely grown aerobically in Luria–Bertani (LB) medium at 30 °C, and 100 μg/ml ampicillin was added to the LB medium for selective cultures.

### Molecular cloning and sequencing

The expression vector pHsh was linearized by inverse PCR using a pair of primers with sequences: 5´-AGCGATAGCAGTTTTTTTCATGGGTATATCTCCTTCTTGT C-3´ upstream, and 5´-GTATCTAGACACCACCACCACCACCACTAATAA-3´ downstream. The PCR product was digested by restriction enzyme *Xba* I such that the linear vector had a blunt end upstream of the target gene, and a sticky end of *Xba* I that was ligated to the 3´-end of a target gene with a fused His-tag.

Genomic DNA was isolated from *Tne* cells according to the instruction of kit (Takara). According to the annotation of the genomic sequence of *Tne* (accession No. CP000916.1), the genes CNT_0580, CTN_1655, and CTN_1756 are open reading frames coding for three putative iron-containing Adhs (Fe-Adh), while CTN_0257 and CTN_1548 are genes encoding a zinc-containing Adh (Zn-Adh) and an Aldh, respectively. These genes were amplified by PCR using Pyrobest DNA polymerase (Takara) with the primers listed in Table 1 and synthesized by Sangon Co. (Shanghai, China). PCR products were digested by *Xba* I and ligated to pHsh vector to yield expression plasmids.

**Table 1:** Primer sequence used in PCR amplification of genomic genes.

Primer names	Primer sequence (5′-3′)
CTN_0580F	TTCAAAATATCATGTTATCTTCC
CTN_0580R	GGTTCTAGAGTAACACCTTCTGAATATCTC
CTN_1548F	AAAATGCTGGTTGCGGGCAGATG
CTN_1548R	GGTTCTAGACTCCCTCAAATCAAAGATGAC
CTN_0257F	AAGGCGGTGAGGCTTCATGCAAA
CTN_0257R	GGTTCTAGACTCGTTCACCATGGTAACCTT
CTN_1655F	GGGAACATGTGGGAGTTCTACAT
CTN_1655R	GGTTCTAGACACACTCAGGGCCTCCCTGTA
CTN_1756F	ATGGAGAATTTCGTCTTTCACAA
CTN_1756R	GGTTCTAGATTTTGCGGCTATTTTCAATAT

### Gene expression and enzyme purification

*E. coli* JM109 cells were transformed using the expression plasmids containing the Adh or Aldh genes and were grown at 30 °C to an OD_600_ of 0.6–0.8 before the cultures were transferred to a 42 °C shaking water-bath incubator for heat-shock induction of gene expression. After 6 h of cultivation along with gene expression at 42 °C, cells were harvested and transferred into an anaerobic chamber for the isolation and purification of recombinant enzymes. *E. coli* cells were re-suspended in degassed 3-(N-morpholino) propanesulfonic acid buffer (MOPSb, 25 mM, pH 6.5), and disrupted by sonication, followed by heat treatment at 75 °C for 30 min.

After the cell debris and heat denatured protein were removed by centrifugation (14,000×*g*, 30 min), the supernatants were mixed with the same volume of 2 × binding buffer, and loaded to the Ni-metal affinity column (Novagen) according to the manufacturer’s instructions. Each recombinant enzyme bound to Ni-column was eluted, and collected into a dialysis bag, concentrated by embedding the dialysis bag in polyethylene glycol (PEG) 20,000, and dialyzed against MOPSb with two changes. Purified enzymes were stored in MOPSb with the addition of 10% glycerol, 1 mM dithiothreitol (DTT), 0.02% (w/v) Sodium azide (NaN_3_), and 1 mM FeCl_2_ for the Fe-containing enzyme.

Gene expression levels and the purity of recombinant proteins were examined using sodium dodecyl sulfate–polyacrylamide gel electrophoresis (SDS-PAGE) as described by Laemmli^15^. Protein concentration was determined by measuring the absorbance at 280 nm and calculating the concentration based on the extinction coefficient for the amino acid sequence of each enzyme.

### Enzyme activity assay

Aldh and Adh activities were measured by monitoring the changes in NAD(P)H absorbance at 340 nm. One unit of enzyme activity was defined as the amount of enzyme producing or consuming 1 μmol of NAD(P)H per min. All the activity assays were performed in triplicate, and data reported here are the mean of the three replicates. For standard enzyme activity assays, the reaction mixture consisted of 100 μl of MOPSb containing suitable substrates at concentrations: 1 mM NAD(P)^+^ or NAD(P)H, 2 mM ac-CoA or CoASH, and 20 mM acetaldehyde (ac-ald) or ethanol, at different pH and temperatures as indicated for each reaction. The CoA-dependent Aldh activity was determined as described by Pei et al.^8^, and the CoA-independent Aldh activity was measured using 20 mM ac-ald as the substrate and 1 mM NAD(P)^+^ as the cofactor, in the absence of CoASH, at 85 °C and various pH values. The *K*_m_ and *V*_max_ of Fe-AAdh on ac-CoA were determined at 80 °C, pH 6.5 with 0.25 mM NADPH as a cofactor. Thermostability of the enzyme encoded by CTN_0580 was analyzed by measuring the residual activity under the optimal assay conditions after pre-incubating aliquots of the purified enzyme for different durations (0 min, 30 min, 60 min, 90 min, 120 min) at 85 °C, 90 °C, and 95 °C.

### Analysis of the protein sequence of Fe-AAdh

The multiple sequence alignment (MSA) of Fe-AAdh, AdhB, and AdhE was used the program T-Coffee and ESPript (Easy Sequencing in PostScript, http://espript.ibcp.fr/ESPript/ESPript/index.php)^16,17^. Similar sequence of Fe-AAdh was also blasted by “Protein BLAST” tool in NCBI (https://blast.ncbi.nlm.nih.gov/Blast.cgi).

### Ethical approval

Not applicable, since the work does not involve any study with human participants or animals.

## Results

### Gene cloning, expression, and enzyme purification

**Figure 1:**
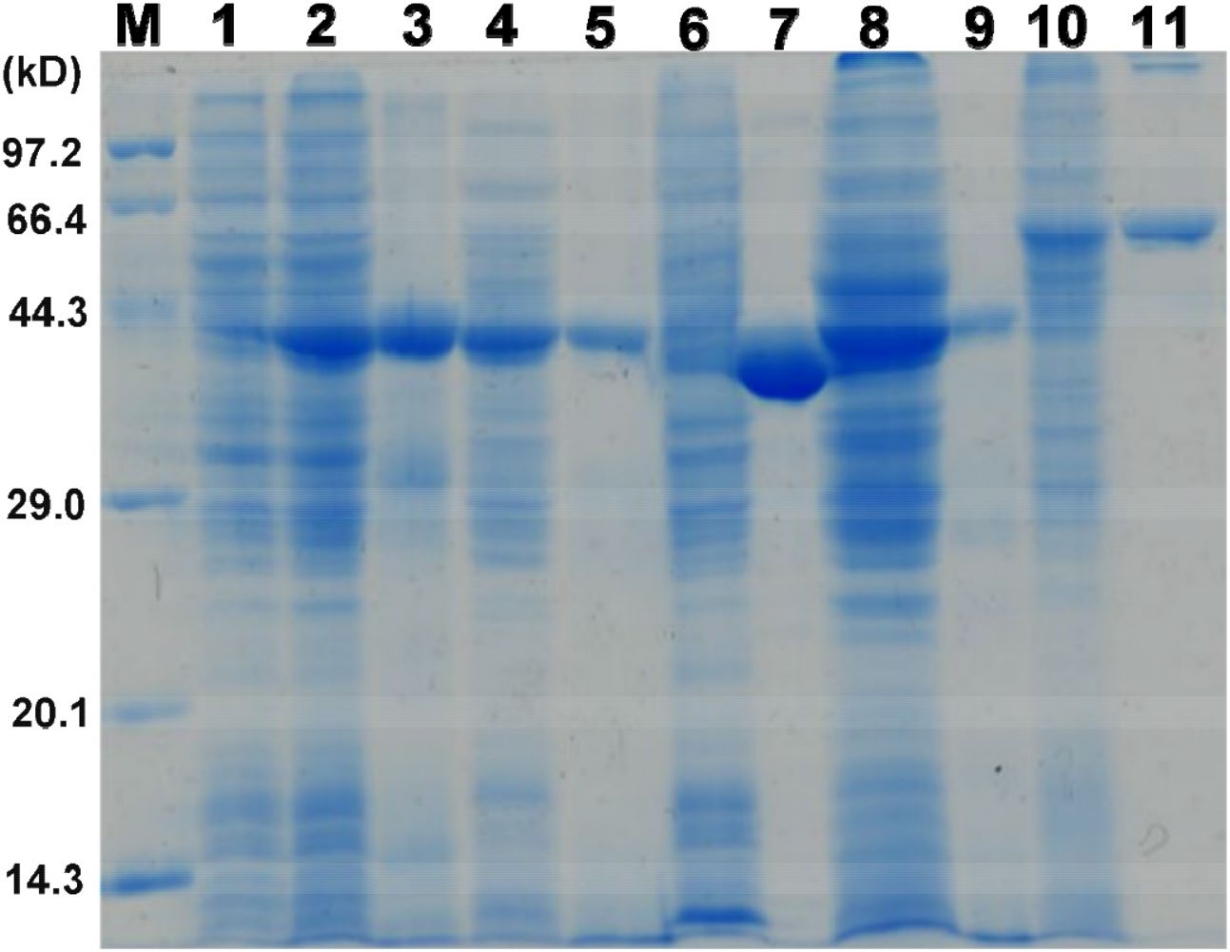
Heterologous expression of the genes encoding aldehyde and alcohol dehydrogenases of *Thermotoga neapolitana*. Lane M, protein markers with molecular masses as indicated; Lane 1, *Escherichia coli* cell protein as control; Lanes 4–5: Fe-AAdh gene (CTN_0580) expressed in *E. coli*, and purified Fe-AAdh protein; Lanes 10–11, protein of *E. coli* cells expressing Aldh gene (CTN_1548), and the purified protein of Aldh; Lanes 2–3, and 6–9: gene expression and purification of the three monofunctional alcohol dehydrogenases (CTN_0257, CTN_1655, CTN_1756).

To prepare recombinant enzymes encoded by the *Tne* genes CTN_0257, CNT_0580, CTN_1548, CTN_1655 and CTN_1756, these genes from *Tne* genomic DNA were amplified, and successfully cloned into *E. coli* expression vector pHsh to generate the corresponding plasmids, i.e. pHsh-0257, pHsh-0580, pHsh-1548, pHsh-1655, and pHsh-1756, respectively. The target genes in the expression plasmids were sequenced and it was confirmed that no mutation had occurred in them. The expression plasmids were transformed into *E. coli* and the target genes were successfully induced to express in the recombinant cells separately (Fig. 1).

The recombinant proteins produced from *Tne* genes CTN_0257, CNT_0580, CTN_1548, CTN_1655, and CTN_1756 were designated as Zn-Adh, Fe-AAdh, Aldh, Fe-Adh1, and Fe-Adh2, respectively, based on their activities and metal ions, and the gene products had subunits similar to their putative molecular masses of 43.3, 42.6, 51.8, 40.6 and 43.6 kD, respectively (Fig. 1). These enzymes were purified to near gel electrophoresis homogeneity after a heat treatment and a metal affinity chromatography by using Ni-column at room temperature (Fig. 1). However, the purified Fe-AAdh became inactivated within about a week after it was stored at − 20 °C, implying that the enzyme was sensitive to O_2_ in the air and buffer. This problem was resolved by purifying these enzymes in an anaerobic chamber using degassed buffers and keeping the purified enzyme in sealed anaerobic serum tubes. The enzyme concentration is about 1.8 mg/mL after purification.

### Dependence of enzyme activity on the temperature and pH conditions

Four possible reactions can occur in the final steps of ethanol formation from ac-CoA. To determine the number of steps each dehydrogenase can catalyze, the biochemical properties of the enzymes were characterized at different reaction pH and temperatures, where substrate(s) and cofactor concentrations were approximately 10 times higher than the *K*_m_ value of the enzyme. *Tne* Aldh encoded by gene CTN_1548 catalyzed the oxidation of aldehydes to acetic acid using either NAD^+^ or NADP^+^ as coenzyme, while Zn-Adh, Fe-Adh1 and Fe-Adh2 did not show any ac-CoA reduction activity (Data not shown in this paper). Therefore, the main biochemical properties of recombinant enzyme encoded by *Tne* gene CNT_0580 were further characterized in more detail.

**Figure 2:**
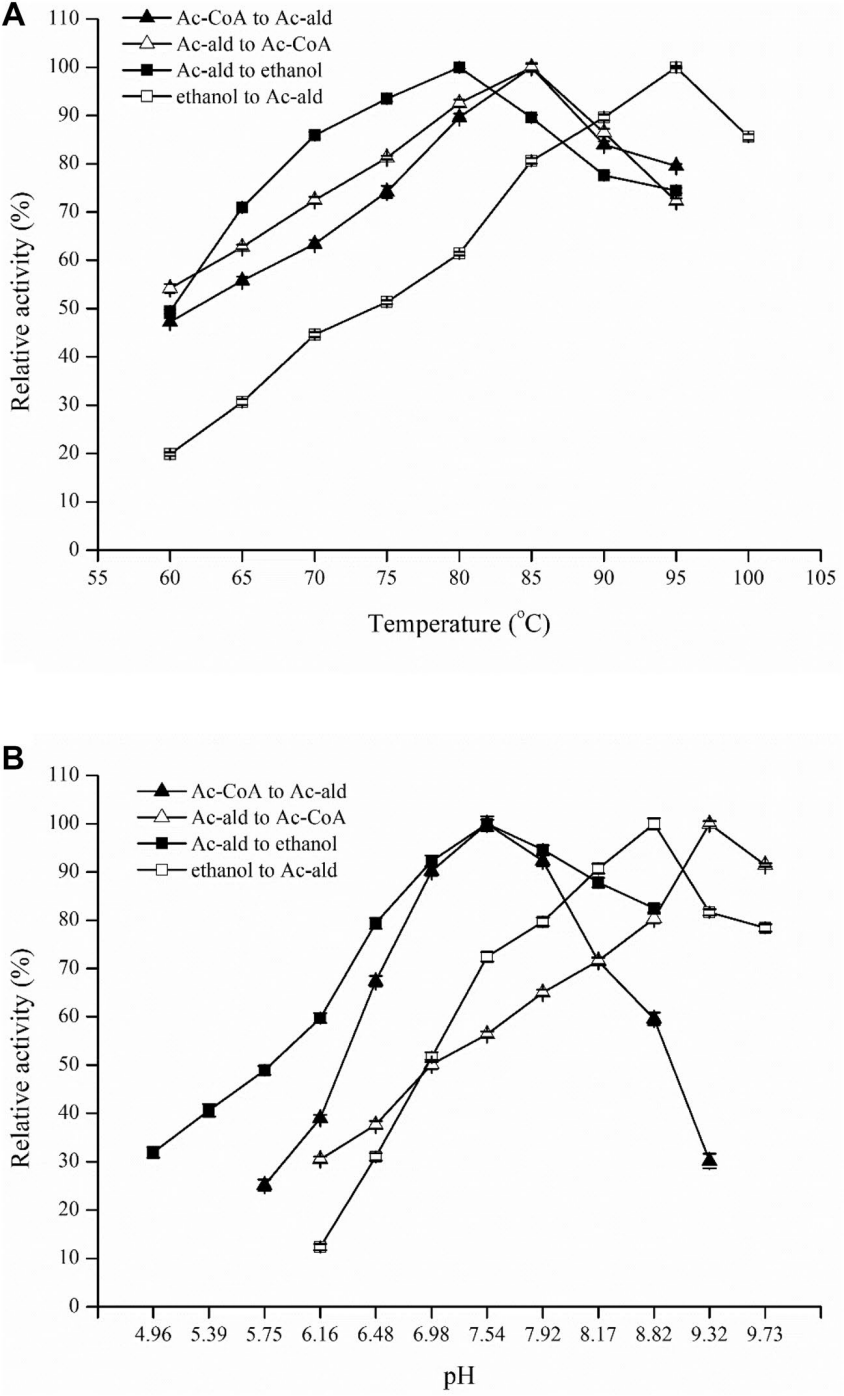
Properties of the bifunctional aldehyde/alcohol dehydrogenase (Fe-AAdh) of *Tne*. (**A**) The effects of temperature on the activities of Fe-AAdh. Activity was determined at various temperatures in the standard reaction mixture described in Materials and Methods. (**B**) The effects of pH on the activities of Fe-AAdh. The buffer composition for these assays was 25 mM each of phthalate, MOPS, Tris, and glycine with pH values calibrated at 80 °C, and the other conditions were the same as those for the standard assays. The highest activity was set as 100%.

When searching for an enzyme that could reduce ac-CoA to ac-ald, we found that the purified enzyme encoded by CNT_0580 was a bifunctional aldehyde/alcohol dehydrogenase (Fe-AAdh). At temperatures up to 95 °C, Fe-AAdh exhibited significant activities in all the reduction and oxidation reactions in the pathway between ac-CoA and ethanol, including the reactions of ac-CoA → ac-ald, ac-ald → ethanol, ethanol → ac-ald, and ac-ald → ac-CoA (Fig. 2A). Fe-AAdh activities highly depended on the temperature and pH conditions, and the enzyme had different temperature and pH optima when it catalyzed these four reactions (Fig. 2). The enzyme exhibited the highest activity for the reductions of ac-CoA and ac-ald at 85 and 80 °C, pH 7.54 (Fig. 2). However, its temperature and pH optima for the oxidations of ethanol or ac-ald were at 95 °C, pH 8.82, or at 85 °C and pH 9.32, which could be much higher than those for growing conditions of *Tne* (Fig. 2).

### Thermostability of Fe-AAdh

**Figure 3:**
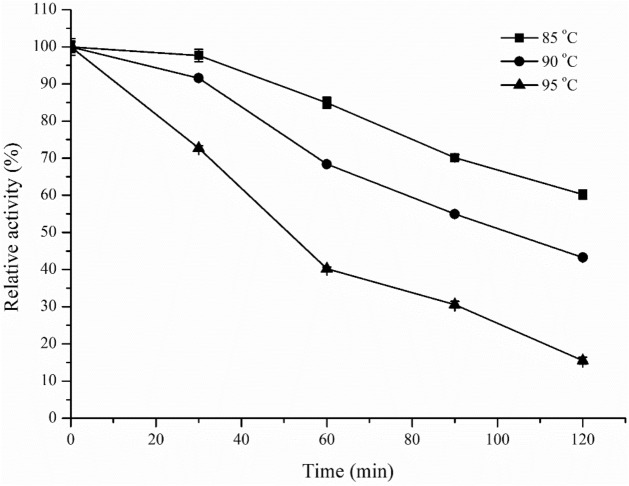
Thermostability of Fe-AAdh catalyzing ac-CoA to ac-ald. After the enzyme was incubated for different times in the absence of substrates, the residual aldehyde dehydrogenase activities were determined by using the standard method. The initial activity was defined as 100%.

Since its gene was cloned from the genomic DNA of *Tne*, a hyperthermophilic strain of *Thermotoga*, Fe-AAdh should be a hyperthermophilic enzyme, which was determined by thermostability of the enzyme (Fig. 3). After Fe-AAdh was incubated at 85, 90, or 95 °C for about one hour, it remained about 85%, 70% or 40% activity in comparison with the original activity without preincubation. Therefore, Fe-AAdh had a 1-h half-life at about 92 °C, which is a typical property of hyperthermophilic enzymes, and the highest among CoA-dependent Aldhs (Fig. 3).

### Cofactor dependence and kinetics of Fe-AAdh

When Fe-AAdh catalyzes the reduction of ac-CoA or ac-ald, reduced type of cofactors NADH or NADPH are involved in the reactions, and on the other hand, oxidized type of cofactors NAD^+^ or NADP^+^ are required for the oxidation of ethanol or ac-ald. The involvements of different cofactors resulted in Fe-AAdh activity changes in each catalytic reaction (Table 2). Fe-AAdh employed NADPH as a sole active cofactor, and no enzyme activity was detected by using NADH in catalyzing ac-CoA reduction (Table 2). The enzyme could use both cofactors in the other three reactions; however, the activities were much higher when using NADPH or NADP^+^ than using NADH or NAD^+^ as a cofactor. Therefore, NADPH and NADP^+^ are preferentially utilized cofactors of Fe-AAdh, and its specific activities for the reductive reactions was significantly higher than those of the oxidative reactions. Although Fe-AAdh showed activity and thermostability at temperatures up to 95 °C, *Tne* cells grew optimally at 80 °C with an intracellular pH value of about 6.5 as measured upon non-buffered cell-free extracts in this study. Therefore, the kinetics of Fe-AAdh was determined under these conditions in the presence of 0.25 mM NADPH as a cofactor. The apparent *K*_m_ and *V*_max_ of Fe-AAdh for ac-CoA were 1.75 mM and 0.79 U/mg, respectively.

**Table 2:** Optimal reaction conditions and coenzyme specificity for enzyme Fe-AAdh.

Enzyme (gene)	Fe-AAdh (CTN_0580)			
Reaction	Ac-CoA → Ac-ald	Ac-CoA ← Ac-ald	Ac-ald → ethanol	Ac-ald ← ethanol
pH_opt_	7.54	9.32	7.54	8.82
T_opt_ (°C)	85	85	80	95
**Specific activity (U/mg)**				
NADPH	0.92 ± 0.03		3.27 ± 0.05	
NADH	ND		0.52 ± 0.06	
NADP^+^		0.41 ± 0.01		2.44 ± 0.04
NAD^+^		0.16 ± 0.02		0.36 ± 0.05

*Ac-CoA* acetyle-CoA, *Ac-ald* acetaldehyde, *ND* activity not detectable.

### Bioinformatic analysis of Fe-AAdh protein

**Figure 4:**
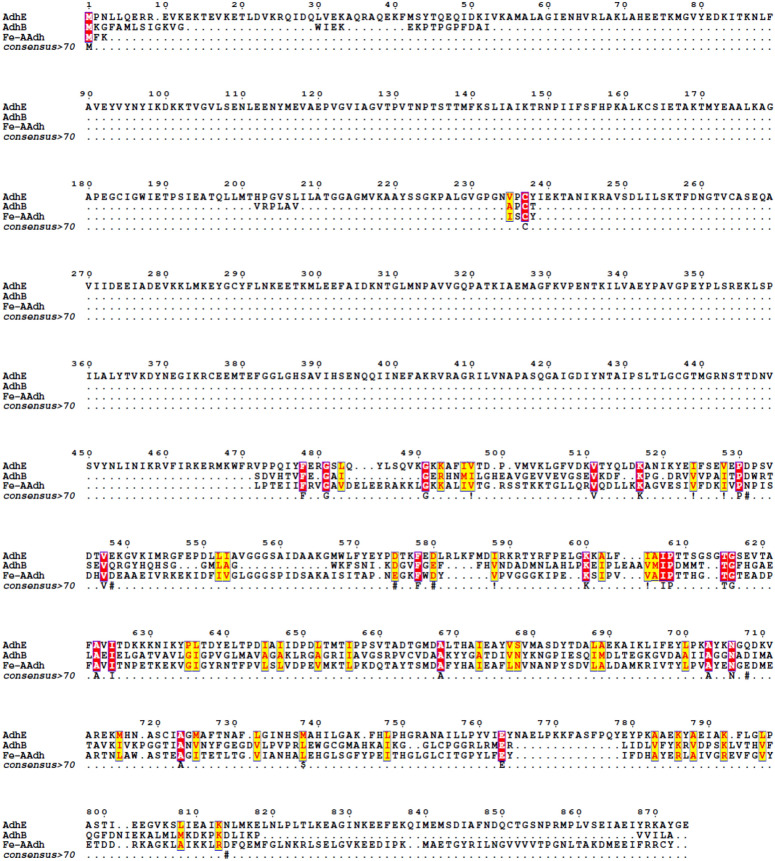
The multi-sequence alignment of bifunctional aldehyde/alcohol dehydrogenases. The domain from residue 30 to 412 of AdhE is related to the aldehyde dehydrogenase family (ALDH-SF superfamily), the domain from residue 551 to 854 belongs to Fe-alcohol dehydrogenase family (PF00465).

The amino acid sequence of Fe-AAdh was subjected to the multiple sequence alignment (MSA) to compare with the other two bifunctional aldehyde/alcohol dehydrogenases, AdhB (GenBank accession number: DQ323135.1) and AdhE (GenBank accession number: DQ836061.1). Interestingly, AdhE is a large protein with two domains of Fe-ADH (PF00465 family) and an ALDH (ALDH-SF superfamily) while bifunctional enzymes of Fe-AAdh and AdhB are monofunctional ADHs (Fig. 4). Meanwhile, Fe-AAdh and AdhB belong to the iron- and zinc-containing alcohol dehydrogenase families, respectively. These results indicate that these enzymes (Fe-AAdh, AdhE, and AdhB) employ different strategies to achieve CoA-dependent aldehyde dehydrogenase activities.

Surprisingly, some similar enzyme also appeared not only in hyperthermophilic *Thermotoga* spp., but also in *Pyrococcus furiosus* (*Pfu*) and *Thermococcus kodakarensis* (*Tko*) (Table 3), which have been just annotated as iron-containing alcohol dehydrogenase (ADH). Thus, basing on these results, the CoA-dependent Aldh activity is probably widespread in hyperthermophilic microbes including bacteria and archaea that grow optimally at temperatures above 80 °C.

**Table 3:** Distribution of Fe-AAdh in some hyperthermophiles.

Microorganism	GenBank No	Pfam family	Sequence similarity (%)
*Thermotoga neapolitana* DSM 4359	CTN_0580	PF00465 (Fe-ADH)	100
*Thermotoga maritima* MSB8	TM_0111	PF00465 (Fe-ADH)	100
*Thermotoga sp.* RQ7	TRQ7_04415	PF00465 (Fe-ADH)	100
*Thermotoga petrophila*	Tpet_0813	PF00465 (Fe-ADH)	99
*Pyrococcus furiosus* DSM 3638	PF_0075	PF00465 (Fe-ADH)	94
*Thermococcus kodakarensis* (ATCC BAA-918)	TK_RS07830	PF00465 (Fe-ADH)	93

## Discussion

Thermophilic fermentation at temperatures above the boiling point of ethanol could allow in situ product removal to mitigate ethanol toxicity. Metabolic engineering is an effective approach to construct novel ethanol-producing strains by heterogeneous expression of the genes coding for the enzymes catalyzing reactions involved in ethanol formation^18,19^. To construct a hyperthermophilic strain for cellulosic ethanol fermentation, researchers have to identify and obtain hyperthermophilic enzymes involved in the ethanol fermentation pathway. The bifunctional POR/PDC has been found in *Tma* and *Thy,* as well as in the hyperthermophilic archaeon *Pfu*. However, POR/PDC can use about 2% and 98% pyruvate to produce ac-ald and ac-CoA, respectively^11,12^. Thus, more than 98% of pyruvate is probably converted to ac-CoA by POR, and ac-CoA is further converted to acetic acid in the absence of an enzyme that converts ac-CoA to ac-ald and further to ethanol.

Basing on the previous studies on the ethanol fermentation pathway of anaerobic thermophiles, the CoA-dependent Aldh activity is usually performed by the enzymes annotated as alcohol dehydrogenases such as AdhB and AdhE^10,20^. There is a gene in *Tne* genome annotated as Aldh, but it was confirmed in this work that the gene product was a CoA-independent Aldh (Data not shown). Therefore, it is necessary to characterize all the enzymes encoded by the Adh genes in a hyperthermophilic microbe.

**Figure 5:**
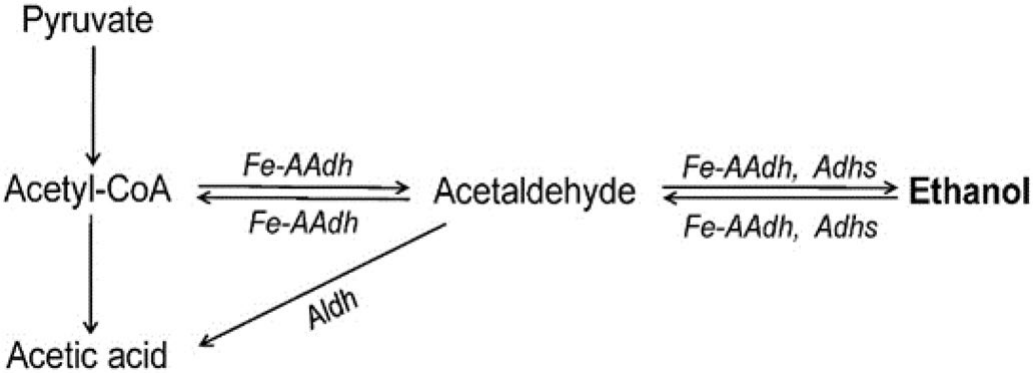
Schematic diagram showing Fe-AAdh and Aldh activities on the reactions in ethanol fermentation pathway of *Tne*.

In the work reported here, a bifunctional aldehyde/alcohol dehydrogenase Fe-AAdh encoded by CTN_0580 was discovered, which could catalyze four redox reactions involved in ethanol formation (Fig. 2A,B). In comparison, Fe-AAdh displayed properties different from those of the other bifunctional ethanol fermentation enzymes, AdhE has very high ac-CoA reduction activity, however, it loses more than 50% activity after an incubation of 30 min at 70 °C and pH 6.5^8^. AdhB from *Tet* is a thermostable enzyme that has a 1-h half-life at 85 °C, however, its specific activity for ac-CoA reduction is below 0.1 U/mg under cell growth conditions. In comparison, Fe-AAdh from *Tne* exhibited the high thermostability of a hyperthermophilic enzyme with 1-h half-life at about 92 °C (Fig. 3). Although the activity of Fe-AAdh in *Tne* in the reduction of ac-CoA is much lower than that of AdhE in *Tet*, Fe-AAdh can be a basic framework for in vitro synthesis of a highly active hyperthermophilic CoA-dependent aldehyde dehydrogenase. This can be achieved by site-directed mutagenesis, motif re-assembly between Fe-AAdh and AdhE, or by directed gene evolution in large scales^21^. Additionally, Fe-AAdh exhibited significant activities to catalyze all four reactions at the optimal reaction conditions (Fig. 5), while AdhE and Adh B can catalyze three reactions for ethanol fermentation^8^.

Many similar genes were also discovered in other hyperthermophiles (Table 3). A gene (PF_0075) encoding an enzyme with 94% similarity to Fe-AAdh from *Tne* was blasted from the genome database of hyperthermophilic archaeon *Pfu*. Previously, a higher amount of ethanol was produced in *Pfu* by co-expression with AdhA or AdhE, which were probably resulted from the synergy between Adh and Fe-AAdh encoded by PF_0075^22,23^.

In conclusion, hyperthermophilic *Thermotoga* spp. are excellent candidates for cellulosic ethanol fermentation. A hyperthermophilic Fe-AAdh was, for the first time, revealed to catalyze ac-CoA reduction to form ethanol via an acetaldehyde intermediate. The Fe-AAdh gene is highly conserved in *Thermotoga* spp. *Pfu*, and *Tko*, indicating the existence of a hyperthermophilic fermentation pathway from ac-CoA to ethanol via ac-ald. These findings offer a basic knowledge for the construction of an ethanol fermentation strain from hyperthermophiles, which is a key element for the approach to the consolidated processes of cellulosic ethanol production.
